# Effect of Sickle Cell Trait on Human Immunodeficiency Virus Type 1 Infection

**DOI:** 10.2174/18746136-v16-e2208150

**Published:** 2022-10-14

**Authors:** Iheanyi Okpala, Chinwe Chukwuka, Seyed Nouraie, Sergei Nekhai, Chima Onwuka, Isa Hezekiah, Onochie Obodo, Deborah Maisamari, Kelechi Okereke, Ajake Oden, Yohanna Tanko, Chinedu Ezekekwu, Vivian Kwaghi, Cajetan Onyedum, Obiageli Nnodu

**Affiliations:** 1 Department of Hematology, University of Nigeria Teaching Hospital, Enugu, Nigeria; 2 Department of Medicine, University of Nigeria Teaching Hospital, Enugu, Nigeria; 3 Department of Medicine, University of Pittsburgh, USA; 4 Department of Medicine and Center for Sickle Cell Disease, Howard University, Washington DC, USA; 5 Department of Hematology University of Abuja, Abuja, Nigeria; 6 HIV Unit, University of Abuja Teaching Hospital, Abuja, Nigeria

**Keywords:** Human Immunodeficiency, Virus Type 1, Sickle Cell Trait

## Abstract

**Introduction:**

Whereas several studies show that homozygous (HbSS) sickle cell disease protects against human immunodeficiency virus infection, it is not clear if human immunodeficiency virus infection is affected by the heterozygous state of the sickle globin gene (HbAS or sickle cell trait).

**Objective:**

To evaluate the effects of sickle cell trait on the prevalence and severity of human immunodeficiency virus type 1 infection in a large patient population.

**Methods:**

Hemoglobin genotype was determined by high performance liquid chromatography (HPLC) in 1,226 HIV-1 patients in Nigeria. Their demographic data were documented. Blood CD4+ cell counts and HIV-1 viral load previously determined on the same blood samples to guide clinical care were used as indices of severity of HIV-1 infection. Statistical analysis of the data was done to evaluate the effects of sickle cell trait on the severity and prevalence of HIV-1 infection, relative to the prevalence of 1.4% in the general population of Nigeria.

**Results and Discussion:**

The distribution of hemoglobin genotypes among the HIV-1 patients was comparable to that in the general population of Nigeria (Chi-squared statistic =1.025; p value = 0.31, not significant). Neither viral load (p = 0.32) nor blood CD4+ cell count (p = 0.30) was significantly different between all HbAS versus all HbAA patients. There was a trend towards lower viral load in females and a significant interaction between gender and HbAS for viral load (P = 0.018), suggesting that sickle cell trait might be associated with the severity of HIV-1 infection in females.

**Conclusion:**

The findings suggest that sickle cell trait might be associated with severity of HIV-1 infection in female, but not all, patients. Larger, prospective studies are required to further investigate the effect of sickle cell trait on HIV-1 infection.

## 1. INTRODUCTION

Homozygous (HbSS) sickle cell disease (SCD) results from inheritance of two sickle globin genes each of which has the nucleotide thymidine instead of the normal adenine (G**A**G => G**T**G) in the sixth codon of beta-globin gene [1]. SCD is characterised by haemolysis of red blood cells which leads to anaemia [2, 3]. Epidemiological and laboratory data strongly suggest that the homozygous HbSS state is associated with significant protection against disease caused by human immunodeficiency virus type 1 (HIV-1) [4 – 8]. Many studies indicate that HIV-1 infection is less prevalent among SCD patients than in the general population [4 – 8]. The prevalence of HIV-1 infection in the general population of Nigeria is 1.4%. Nkiruka David and co-workers found absence of individuals with SCD in a cohort of 208 children receiving care for HIV in Lagos, Nigeria [4]. Among 650 adults with sickle cell anaemia (SCA) registered in University of Nigeria Teaching Hospital, Enugu, Nigeria, none had HIV-1 infection. In 69 pediatric SCD patients who had previous blood transfusion at the same University of Nigeria Teaching Hospital, Ubesie *et al* found only two (2.9%) with HIV infection [9]. No individual with HIV-1 infection was found among 903 patients in the Sickle Pan African Research Registry at the University of Abuja Teaching Hospital, Abuja, Nigeria [10]. These observed figures are significantly lower than the expected SCD prevalence rate of 10–20/1000 in the general Nigerian population. HbAA is the standard notation for the hemoglobin genotype of a person who is homozygous for the normal beta-globin gene in adults (HbA). HbAS is the standard notation for the hemoglobin genotype of a person who has one gene for the normal beta-globin gene in adults (HbA), and one gene for the sickle beta globin (HbS). This heterozygous condition is called sickle cell trait, and the person who has it is called a carrier of the sickle globin gene. HbAC is the notation for the hemoglobin genotype of a person who has one gene for the normal beta-globin gene in adults (HbA), and one gene for the variant hemoglobin C. HbSC is standard notation for the hemoglobin genotype of a person who has one gene for sickle beta-globin (HbS), and one gene for hemoglobin C. This condition is called HbSC disease.

In Nigerian pre-school children, Kaine *et al* reported prevalence rates of 22.5% for HbAS, 1.6% HbSS and 75.8% HbAA [11]. In a cohort of 3,603 newborns from Gwagwalada Area Council, Nigeria, Nnodu *et al* found prevalence rates of HbAA 77.0%, Hb AS 20.5%, HbSS 1.4%, HbAC 0.9%, and HbSC 0.1% [12]. The 2018 Nigerian National Demographic Health Survey of 11,243 children of ages 6–39 months found prevalence rates of 77.8% HbAA, 19.9% Hb AS, 0.9% Hb SS, 1.4 HbAC, 0.3% Hb SC, 0.1% Other genotypes, which include HbA/Betathalassaemia and HbA/Beta^+^thalassaemia genotypes [13].

Consistent with the findings in Nigeria, several studies in USA found significantly reduced prevalence of HIV infection in SCD patients compared with people without this haemoglobinopathy [5 – 8]. Castro and colleagues found no HIV positive person in a cohort of 116 SCD patients, 88 of whom had received a mean of 18.6 transfusions of red blood cells [5]. Nouraie’s team observed that SCD is associated with reduced HIV-1 diagnosis but higher hepatitis B virus (HBV) and hepatitis C virus (HCV) infection among approximately 500,000 US hospital patients [6]. Bagasra and co-workers observed that long-term non-progression of HIV-1 infection to AIDS in US patients is significantly associated with SCD [7]. Kelly *et al* observed a significantly lower risk of HIV infection in people with SCD compared to patients with non-SCD congenital anemia (Odds Ratio 13.1, 95% CI 1.6–108.9) [8]. Molecular studies by Nekhai’s group demonstrated restriction of ex vivo HIV infection of peripheral blood mononuclear cells (PBMCs) obtained from SCD patients in association with alterations in inflammatory cytokines, regulation of iron metabolism and hypoxia-related host factors [14, 15].

In contrast to the many studies that observed protective effects of homozygous (HbSS) SCD against HIV-1, comparatively fewer clinical or epidemiological studies have demonstrated that sickle cell trait (SCT, the heterozygous HbAS state) confers benefit to people infected by HIV-1 [4, 14]. It is also not certain whether the heterozygous HbAS state is protective against acquisition of HIV-1 infection. This study evaluated the effects of SCT on the prevalence and severity of HIV-1 infection in a large population of 1,226 HIV-1 patients who receive care at the University of Nigeria Teaching Hospital, Enugu; and University of Abuja Teaching Hospital, Abuja; both in Nigeria.

## 2. MATERIALS AND METHODS

Following Institutional Review Board approval and participants’ informed consent, high performance liquid chromatography (HPLC) was used to determine the hemoglobin genotype of 1,226 persons who have HIV-1 infection [16]. These included 816 patients from University of Nigeria Teaching Hospital, Enugu; and 410 patients from University of Abuja Teaching Hospital, Abuja, Nigeria. All patients with HIV-1 infection receiving care in both centers who gave informed consent were recruited. All the HIV patients were on antiretroviral therapy regimen that includes tenofovir, lamuvidine and dolutegravur or efavirenz. In all patients, the HIV infection was at the stage that requires treatment. The patients’ demographic data collected included age and gender. Peripheral blood CD4^+^ cell counts and HIV-1 viral load previously determined at diagnosis to guide clinical care of the patients were used as indices of severity of the HIV-1 infection. The collated data were subjected to statistical analyses to evaluate the effects of sickle cell trait (HbAS genotype) on the prevalence and severity of HIV-1 infection. The minimum sample size of participants who have sickle cell trait (HbAS genotype) required for the study was determined using the formula N = Z^2^ × p (1-p) /alpha^2^, where N is the minimum sample size, Z is the standard deviation at 95% confidence interval (1.96), p is the prevalence of sickle cell trait in Nigeria (0.23), and alpha is the margin of error (0.05). Therefore, N = 1.96^2^ × 0.23 × 0.77/0.05^2^ = 226 people who have sickle cell trait. To make allowance for patients who might have incomplete data for some parameters to be analysed, as would be expected in a retrospective study, data from 258 HbAS patients with HIV-1 infection were collated. The viral load and CD4+ve T-cell count were done at the stage of HIV infection when the patients needed antiretroviral therapy. The CD4+ve T-cell count and viral load data used for each participant were obtained from blood samples taken at the same time point. However, the two tests were reported on separate laboratory results documents. The study laboratory data were collected retrospectively, and information on specific parameters was not available in some participants’ medical records. This led to the number of participants who had viral load data being different from the number whose CD4 +ve T-cell count was available.

Statistical Analyses: The Chi-Squared test was used to find out if there is a significant difference in the proportions of people who have sickle cell trait among HIV-1 patients in this study and the general population of Nigeria.

## 3. RESULTS

The 1,226 HIV-1 positive patients included 879 females and 347 males of age range 5–82 years, median age 44 yrs, and inter-quartile range 38–52 yrs.

### Distribution of hemoglobin genotypes in HIV-1 patients

The hemoglobin genotype was normal HbAA in 965 (78.7%) of the patients, heterozygous HbAS or sickle cell trait in 258 (21.%) and other in 3 (0.3%). The percentages of people with hemoglobin genotypes AA and AS among the 1226 HIV-1 patients are comparable with prevalence rates in the general Nigerian population, Table 1 [13 – 15].

The difference between 100% and the sum of the HbAA and HbAS percentages in HIV-1 patients and the general Nigerian population is the percentage of other hemoglobin genotypes which are HbA/Beta Thalassaemia and HbAC genotypes.

### Viral load and blood CD4^+^ cell count in HIV-1 patients

HIV-1 viral load determined to guide clinical care were obtainable from the hospital records of 732 HbAA and 201 HbAS patients. Peripheral blood CD4^+^ cell counts previously done for the same purpose were obtainable from the hospital records of 932 HbAA and 245 HbAS patients. Indices of severity of HIV-1 infection, viral load and blood CD4^+^ cell counts were compared between HbAA and HbAS individuals with HIV-1 infection (Table 2). No statistically significant differences in viral load and blood CD4+ cell counts were detected when *all* HbAA patients were compared with *all* HbAS persons.

However, there was a trend towards lower viral load in females (Table 3), and significant interaction between gender and HbAS genotype for viral load (P = 0.018), suggesting that sickle cell trait might be associated with severity of HIV-1 infection in women.

## 4. DISCUSSION

The results of this retrospective analysis of available data on HIV-1 viral load and peripheral blood CD4^+^ cell counts in the hospital records of a large population of Nigerian patients whose hemoglobin genotype was prospectively determined during the study suggest that, relative to the normal hemoglobin genotype AA, the heterozygous HbAS condition or sickle cell trait does not significantly affect the prevalence nor severity of HIV-1 infection if *all* the HIV-1 patients who have normal HbAA genotype are compared with *all* HIV-1 patients who have sickle cell trait (HbAS genotype). However, there was a trend toward lower viral load in HbAS females suggesting that sickle cell trait might be associated with severity of HIV-1 infection in women, but not in all persons living with HIV-1. In the light of this, it might be informative to further evaluate the effect of sickle cell trait on HIV-1 infection in larger, prospective, multi-center studies.

The mechanism(s) of the protective effect of homozygous HbSS state or sickle cell disease on HIV-1 infection are not fully elucidated. Nekhai *et al*, and other investigators, have shown that reduction of cellular iron by iron chelators inhibits HIV-1 replication [14, 15]. Cellular iron is exported by ferroportin (FPN), a membrane protein, which is negatively regulated by hepcidin secreted by the liver. During the acute phase of HIV-1 infection, levels of plasma hepcidin are increased and levels of plasma iron are decreased. During the later phases of HIV-1 infection, levels of hepcidin remain elevated and positively correlate with viral load, both in untreated infection and that treated with antiretroviral therapy [14, 15]. Thus, iron metabolism affects HIV-1 infection and disease progression. Nekhai and co-workers showed that HIV-1 transcription is inhibited in cells that express FPN, HIV-1 is upregulated in T cells treated with hepcidin, and that peripheral blood mononuclear cells from sickle cell disease patients which express FPN show inhibition of HIV-1 reversed by hepcidin [14, 15]. These findings indicate FPN-mediated iron export and HIV-1 inhibition in sickle cell disease.

It is not clear how HIV load might be associated sickle cell trait in women but not in all persons who have this viral infection, and prospective studies are required not only to confirm this, but also elucidate the mechanisms.

## CONCLUSION

We evaluated the effect of sickle cell trait (HbAS) on HIV-1 infection in a large population (1,226) of HIV-1 patients in Nigeria. The prevalence of sickle cell trait in HIV-1 patients was comparable to that of the general population of Nigeria. The data suggest that HbAS females have lower HIV load than their HbAA counterparts, but this, and the overall effect of sickle cell trait on HIV infection, needs to be further evaluated in prospective studies because new knowledge so obtained might be relevant to clinical care.

## Figures and Tables

**Table 1.** Chi-Squared Test of the difference between the proportions of people with sickle cell trait in Nigerians who have HIV-1 infection and the general Nigerian population [11].

Hb Genotype	Nigerians with HIV-1 Infection	General Nigerian Populatio	Row Totals
HbAA	965 (78.7%)	758 (75.8%)	1723
HbAS	258 (21.0%)	225 (22.5%)	483
Column Totals	1233	983	
Grand Total 2206			

Chi-squared statistic =1.025 p value = 0.311(not significant).

**Table 2.** HIV-1 load and blood CD4^+^ cell count in HbAA versus HbAS individuals

	HbAA Genotype	HbAS Genotype	P value
Viral load: median (IQR) copies /ml of blood	20 (0–66) [N = 732]	20 (0–66) [N = 201]	0.32
Patients with detectable viral load (>20 copies/ml)	254/732 (34.7%)	70/201 (34.8%)	> 0.9
CD4^+^ count: median (IQR) cells/mm^3^ of blood	527 (353–732) [N = 932]	499 (321–709) [N = 245]	0.30

IQR: Inter-Quartile Range.

**Table 3.** Viral load according to hemoglobin genotype and gender among 933 HIV-1 patients.

	Female		Male		P for interaction
	AA	AS	AA	AS	
Mean (SD) of the natural log of viral load in all patients	5.21 (3.26)	4.54 (2.83)	5.36 (3.49)	6.43 (3.73)	0.018
Number of patients with detectable viral load of >20 copies/ml	189 (34.9%)	48 (33.8%)	65 (34.0%)	22 (37.3%)	0.64

## Data Availability

The data that support the findings of this study are available on request from the corresponding author.

## LIST OF ABBREVIATIONS

HbA: Adult Hemoglobin
HbS: Sickle Hemoglobin
SCD: Sickle Cell Disease
HbSS: homozygous HbS genotype
HbAS: Sickle Cell Trait
HBV: Hepatitis B Virus
HbAA: Homozygous HbA genotype
HBC: Hepatitis C Virus
